# Metallomic Biomarkers in Cerebrospinal fluid and Serum in patients with Parkinson’s disease in Indian population

**DOI:** 10.1038/srep35097

**Published:** 2016-10-18

**Authors:** Jaya Sanyal, Shiek S. S. J. Ahmed, Hon Keung Tony Ng, Tufan Naiya, Epsita Ghosh, Tapas Kumar Banerjee, Jaya Lakshmi, Gautam Guha, Vadlamudi Raghavendra Rao

**Affiliations:** 1 Department of Anthropology, University of Delhi, Delhi 110007, INDIA; 2 Drug Discovery Lab, Faculty of Allied Health Sciences, Chettinad Academy of Research and Education, Tamil Nadu, INDIA; 3 Department of Statistical Science, Southern Methodist University, Dallas, TX 75275, USA; 4 Molecular Systematics Division, Zoological Survey of India, DNA Taxonomy Laboratory, Kolkata, INDIA; 5 Department of Neurology, National Neurosciences Centre, Kolkata, INDIA; 6 Dept. of Life Sciences, Central University of Tamil Nadu, Thiruvarur, Tamil Nadu, INDIA; 7 Department of Neurology, Nil Ratan Sircar Medical College and Hospital, Kolkata, INDIA; 8 Dept of Genetics, Osmania University, Hyderabad 500017, INDIA; 9 Genome Foundation, Hyderabad 500076, INDIA

## Abstract

Parkinson's disease (PD) is a neurodegenerative disease with the absence of markers for diagnosis. Several studies on PD reported the elements imbalance in biofluids as biomarkers. However, their results remained inconclusive. This study integrates metallomics, multivariate and artificial neural network (ANN) to understand element variations in CSF and serum of PD patients from the largest cohort of Indian population to solve the inconsistent results of previous studies. Also, this study is aimed to (1) ascertain a common element signature between CSF and serum. (2) Assess cross sectional element variation with clinical symptoms. (3) Develop ANN models for rapid diagnosis. A metallomic profile of 110 CSF and 530 serum samples showed significant variations in 10 elements of CSF and six in serum of patients compared to controls. Consistent variations in elements pattern were noticed for Calcium, Magnesium and Iron in both the fluids of PD, which provides feasible diagnosis from serum. Furthermore, implementing multivariate analyses showed clear classification between normal and PD in both the fluids. Also, ANN provides 99% accuracy in detection of disease from CSF and serum. Overall, our analyses demonstrate that elements profile in biofluids of PD will be useful in development of diagnostic markers for PD.

Parkinson’s disease (PD), the second most common neurodegenerative disorder, characterized by progressive loss of dopaminergic neurons at substantia nigra of the brain. Nearly 6.3 million were affected worldwide, and it is expected to double over the next twenty years^1^. Degeneration of dopaminergic neurons affects motor functions, which include motor initiation, tremor, slowness of movement and other cognitive capabilities. Etiology of the disease is largely unknown, where a combination of known etiology associated PD ranges from genetic predisposition to environment factors. The current diagnosis of PD is carried out by neurological examination and medical history. Further, severity of disease is categorized as stages based on Unified Parkinson’s Disease Rating Scale (UPDRS) or Hoehn and Yahr scale or Schwab and England Activities of Daily Living Scale. However, there is no definitive marker for diagnosis of this disease; thus, an understanding of the molecular basis of disease pathology is highly important. Multi-factorial complexities and lack of molecular markers causes delay in diagnosis^2^. Increased biomarkers diagnostic sensitivity at early phase would enable subjects to therapeutic intervention. In addition, markers from readily accessible biofluids such as saliva, serum, urine or CSF will add feasibility for rapid diagnosis of PD.

Metallomics is a powerful tool that demonstrates perturbations in the trace and ultra-trace elements of cell, tissue and biofluids. Element interacts at various states of biomolecules such as DNA, RNA and protein that represents biochemical phenotype of an organism, in both normal and disease condition. Hence, element analyses were routinely practiced in many diseases to make a valuable decision on pathophysiology. Advancement in analytical techniques facilitates the establishment of complete element profile of a sample providing valuable information on element homeostasis that can be used as biomarkers. Several studies demonstrated the imbalance of essential elements, such as Al, Ca, Fe, Mg, Pb, Fe, Cu and Zn which play a vital role in biological process and have greater association with PD^3^,^4^, whereas other studies have shown a negative association^5^,^6^. Investigations of these studies remain controversial pointing to accumulation as well as depletion of elements in biofluids of PD, possible reasons for which include sample size and differences in ethnicity. In India, very few studies have reported the levels of elements in biofluids of PD. Moreover, these studies were restricted with limited sample size and uses serum and plasma as biofluids for elements level estimation^7^,^8^,^9^. However, their results are inconclusive as they fail to correlate their results with elements levels of CSF, which highly reflects brain-specific changes. Hence, detection of trace elements of serum/plasma that resembles the profiles in CSF will be appreciable for the diagnosis from serum/plasma.

This study is focused to gain an understanding of trace element variations in CSF and serum of PD patients from the largest cohort of Indian population to solve the inconsistent results of previous studies. Here, we systematically quantified levels of 11 trace elements such as aluminium (Al), calcium (Ca), chromium (Cr), cobalt (Co), copper (Cu), iron (Fe), magnesium (Mg), manganese (Mn), lead (Pb), silicon (Si) and zinc (Zn) in CSF and serum of normal and PD patients using atomic absorption spectrophotometry and flame atomic absorption spectrophotometry. Furthermore, we compared CSF element profiles with serum profiles of patients to determine efficient diagnostic biomarkers from serum which reflects pathophysiological mechanisms of the brain.

## Results

We employed targeted element analysis of Al, Ca, Cr Co, Cu, Fe, Mg, Mn, Pb, Si and Zn to determine changes in elements associated with Parkinson’s disease in CSF and serum from patients diagnosed based on neurological examination. The baseline clinical and demographic data of 530 subjects (250 PD and 280 controls) contributed serum for our study are summarized in Table 1. Age and gender distribution of patients and controls were found similar with p-values 0.323 and 0.965, respectively. The mean age of PD onset was 53.1 ± 11.2 years with UPDRS score 31.2 ± 5.2 and H&Y staging scale 2.4 ± 1.1. Also, H&Y scale revealed 127 PD patients (50.8%) in stages 1 and 2, while others are in stage 3 or above. Among 250 patients, 196 were under anti-parkinsonian medications (mPD) including levodopa-carbidopa, or levodopa-carbidopa + ropinirole/pramipexole, while remaining drug naive PD (umPD) were included to determine the confounding effect of the medication. Most of the patients reported an increase in tremor and imbalance during periods of stress. There was no difference in the clinical features of the male and female patients. Further, CSF was collected from 110 individuals, 60 normal and 50 PD (46 were in treatment, 4 were drug naive), patients. The mean (±SD) age of CSF controls was 60.11 ± 10.44 years, while mean age of the patients was 58.72 ± 12.38 years; H&Y staging scale was 2.16 ± 1.09 with duration of disease 3.77 ± 2.3 years (Table 1).

### Elements concentration

For each sample, atomic absorption spectrophotometry and flame atomic absorption spectrophotometry were applied to determine element concentration in CSF and serum of normal and PD patients. Analysis of spectrophotometry data provides element concentration in both the fluids, expressed in μg/lt. Elements detected in CSF and serum of normal was confirmed based on a comparison with known standard and scientific literatures. Heat maps for CSF and serum were generated from the trace elements concentration, revealed differences between control and PD. The elements represented in red color are over expressed, and those elements with green are under expressed (Supplementary Fig. S1). Ca, Cr, Pb and Mg were significantly elevated among PD patients compared to the controls in CSF. In contrast, levels of aluminum, cobalt, iron, manganese, silicon and zinc were significantly reduced in patients compared to healthy control. However, no significant changes were noticed for copper between the groups (Table 2). Further, the analysis of serum samples (Supplementary Fig. S2) revealed significant increase in elements concentration of aluminum, calcium, lead, magnesium and decrease in copper and iron. Elements such as chromium, cobalt, manganese, silicon and zinc do not attain minimum significant variations between normal and PD patients (Table 2). Sub-group analysis of mPD and umPD show no significant changes in elements concentration in both the fluids (Table 3). Interestingly, the analysis based on akinetic-dominant vs. controls showed significant association of increased Mg and decreased Cu, Co in CSF PD (Table 4). On the other hand, the analyses of tremor-dominant showed association of elevated calcium, magnesium, lead, chromium and decreased manganese, silicon, zinc, iron, cobalt levels in CSF (Table 5). Similarly, changes were noticed in serum of akinetic-dominants and tremor-dominants PD patients, which provide the elemental influence on clinical symptoms of PD (Tables 4 and 5).

### Correlation and ratio of trace elements

**Figure 1:**
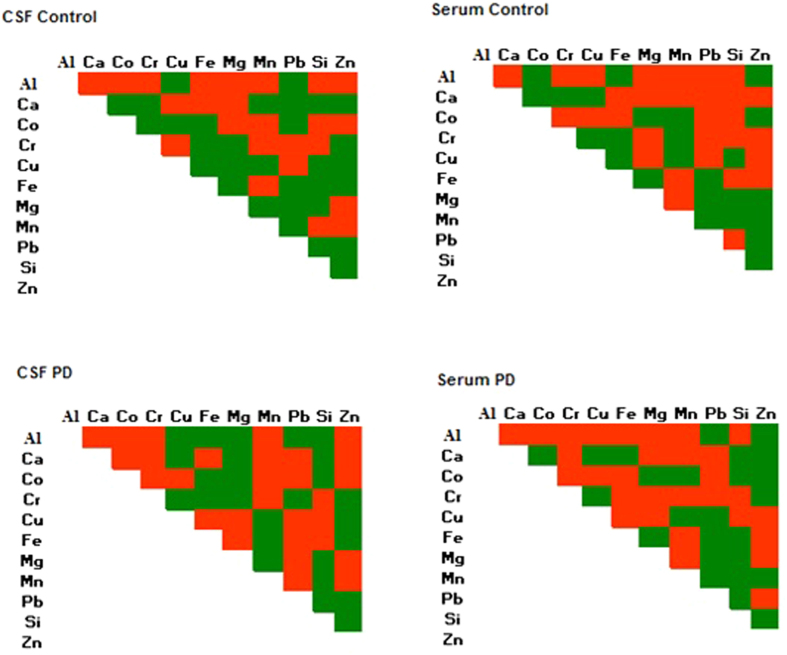
Element-element interaction patterns in CSF and serum among PD Patients and controls.

The interdependency between the elements in the biological fluids was determined using Pearson’s correlation. Of 55 element-element interaction, 25 were positive and 30 were negatively correlated in normal CSF, whereas 30 were positive and 25 negatively correlated in normal serum (Fig. 1). In PD, correlation analysis of CSF and serum showed different interaction patterns when compared with normal CSF and serum (Fig. 1). Altered interaction between Cu-Fe, Cu-Ca, Fe-Mn, Fe-Pb, Fe-Mg, Mn-Ca, Mn-Pb, Pb-Ca, Pb-Cr, Pb-Co, Zn-Ca, Mg-Cu, Mg-Al, Mg-Co, Ca-Cr, Al-Fe, Cu-Co in CSF and Pb-Cu, Fe-Ca, Fe-Cr, Mn-Cr, Zn-Pb, Zn-Ca, Zn-Mg, Zn-Cr, Al-Pb, AL-Fe, Al-Co in serum was noticed. The microgram percentage ratios and correlation patterns (Supplementary Table 1a,b) of the elements indicate that there is an imbalance in the element to element interrelationships among PD affected individuals. In an attempt to evaluate barrier function, metal concentrations in CSF were divided by the corresponding in blood serum to produce an indirect measurement of CSF/blood serum (C/B) ratios (Supplementary Table 2). Mg, Pb, Cr metals showed C/B ratios above the value 1 both for PD cases and controls, in particular, higher concentrations in CSF than in the serum, indicating possible accumulation inside blood-brain barrier. Average ratios for Fe, Cu, Ca, Al, Mn, Si, Zn and Co were below the value 1 i.e. higher concentrations in serum than in CSF, both for PD cases and for controls. Cu showed the lowest ratios both for PD cases and controls. Higher concentrations in serum than in CSF, although with higher C/B ratios among PD cases, were noted for Al. Ca and Cu showed approximately the same average ratios for CSF as for serum whereas, Fe, Mn, Zn, Si and Co have lower ratios among patients. (Supplementary Table 2).

### Multivariate Analysis

**Figure 2:**
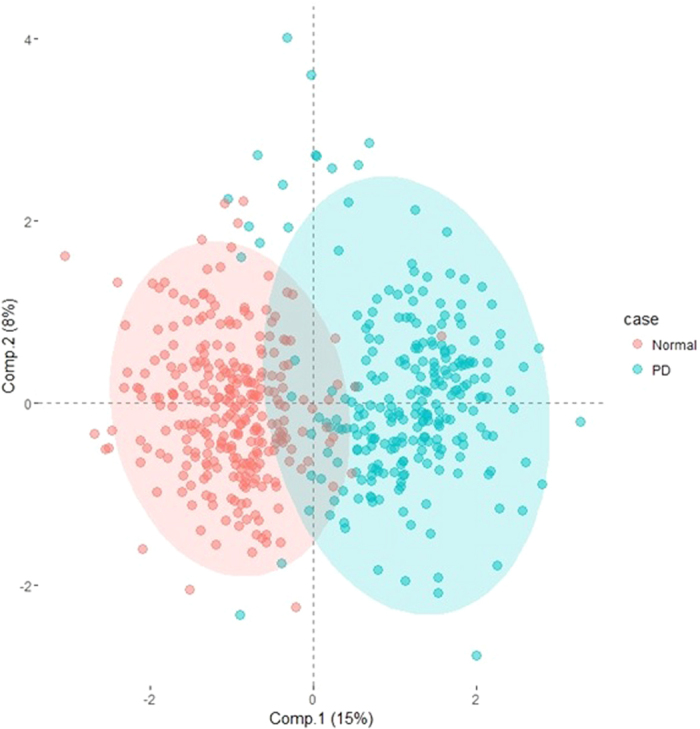
PLS-DA (3 LVs and 1 OSC LVs) scores plot for normal and PD groups in the serum data (R2 = 0.751, Q2 = 0.725). To assess the quality of the computed model, we can compare our model fit to random chance by 11 folded cross-validation.

**Figure 3:**
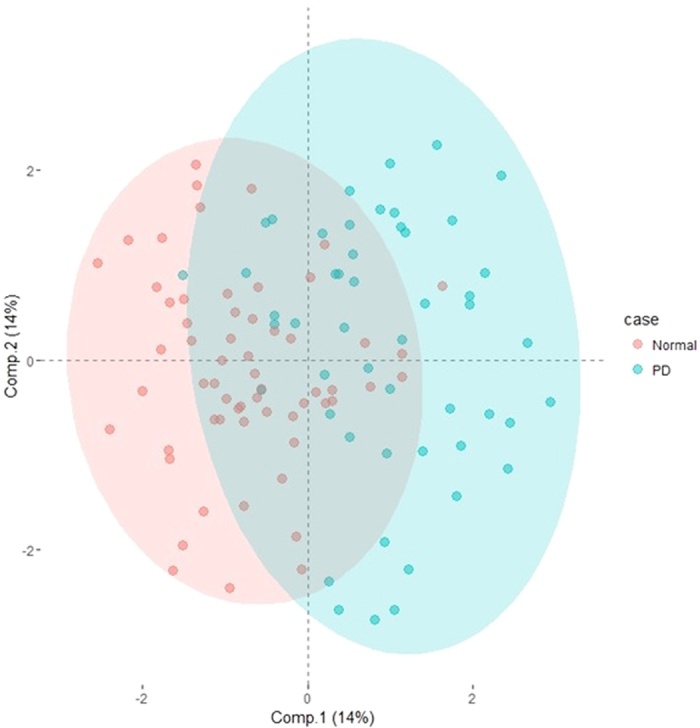
OPLS-DA (2 LVs and 1 OSC LVs) scores plot for normal and PD groups in the CSF data (R2 = 0.521, Q2 = 0.359).

The classification between experimental groups (control, mPD and umPD) was evaluated using multivariate analysis, to determine whether the behavior of trace element data of CSF and serum differs among the subjects. Two distinct classification groups were noticed between control and PD in both CSF and serum (Figs 2 and 3) based on the OPLS-DA. However, no clear discrimination was noticed between treated (mPD) and untreated PD (umPD). To ensure observed grouping and reliability of model, an internal 10-fold cross-validation was performed. The goodness of fit based on the OPLS-DA was calculated as 0.521 for CSF and 0.751 for serum and the goodness of prediction (Q2) was calculated as 0.359 for CSF and 0.725 for serum respectively, which confirms the predictability of the model (Supplementary Figs S3 and S4). The score plots in Figures S3 and S4 show that the resulting models have reasonable ability to separate the difference between the control and PD groups. Despite the discriminanting ability of the two fluids, separation of the control and PD in serum is better than that of CSF.

### Neural network analysis

The classifier algorithms were trained and tested using the trace element profile along with the age and clinical symptoms data such as rigidity, rest tremor, bradykinesia, micrographia, masked faces, dementia, depression, postural imbalance and gait difficulties. The presence of each clinical symptom was represented as ‘‘1’’, where absence denoted as ‘‘0’’. Three network models were created using the classifier algorithms to classify the data set into (1) *Class* (normal and Parkinson’s disease), (2) *Stages* (normal, stage1, stage2 and stage3 PD) and (3) *Status* (normal, progressive and static). The detailed performance of the classifiers were shown in Table 6A,B. In CSF (Table 6A), the BayesNet algorithm performs better compared to other algorithms in classification of data into *Class* (99.78% accuracy) and Stages (71.73% accuracy). For the classification based on disease status, the random forest algorithm showed better accuracy of 84.09% in classifying the disease status as normal, progressive and static. In serum (Table 6B), BayesNet, JRip and Multilayer Perceptron algorithms were determined to be efficient with accuracies of 99.87, 72.95 and 91.07% in classifying the *Class*, *Stages* and *Status*, respectively. Overall, the performance of all the analyzed classifiers was extremely fast and more accurate with minimum accuracy of 60 percentages.

## Discussion

Metallomics is an emerging technique that provides disease-specific fingerprints of perturbations in trace or ultra-trace elements, reflecting the change in molecular mechanism due to disease pathophysiology. Element analysis is routinely carried out in basic clinical laboratories for the diagnosis of many diseases, which is ease and accurate. Analysis of elements in biofluids, such as serum, plasma, urine or CSF is preferred for most of the diagnostic innovations due to ease processing, rapid and cost effective.

Several trace element analyses of Parkinson’s disease have been conducted on serum, plasma and urine. However, inconsistency in the levels of variation in elements among these studies persist, which may be due to limited sample size and population variations. In the present Indian scenario, changes in trace elements mostly remain elusive in PD with the limited number of observations. In addition, most of these studies were conducted in serum/plasma samples, but considering the close proximity to the brain, CSF will be an efficient biofluid that provides valuable information on molecular mechanism and markers for diagnosis. However, extraction of CSF by lumbar puncture, a more invasive procedure, limits acceptance of patients for routine analysis. Hence, the elements levels in serum that resembles the profiles of CSF will aid and ease diagnosis for routine clinical practice.

In this study, CSF samples were obtained from 110 individuals (50 patients and 60 controls) and cross compared with the profiles of 530 serum samples. For subgroup analysis, patients were grouped as drug naive patients and patients under medication. To determine the effect of anti-parkinsonian treatment in the element profile, drug naive patients were compared with an equal number of age-sex matched PD patients under medication selected at random. Comparative subgroup analysis on clinical presentation, such as akinetic-dominant and tremor-dominant were carried out, to determine the elements influence on clinical symptoms (Tables 4 and 5).

To our knowledge, this is the first study carried out in Indian ethnicity with large cohorts, (1) to evaluate the elements changes in normal and PD subjects in both CSF and serum to solve the inconsistent results of previous studies. (2) To establish, the unique and common elements variations in serum and CSF of PD. (3) To demonstrate the elements association with clinical presentations, and (4) to validate serum as a reliable biofluid for elemental studies of PD. The analysis of spectroscopic data explored the significant associations between CSF and serum concentration of trace elements and the risk of Parkinson’s disease, demonstrating that lower concentrations of Aluminium (Al), Cobalt (Co), Copper (Cu), Iron (Fe), Manganese (Mn), Silicon (Si), Zinc (Zn) and higher concentrations of Calcium (Ca), Magnesium (Mg), Lead (Pb), Cromium (Cr) in CSF samples might be associated with disease susceptibility. Similar trend was noticed in serum elemental data. However, Cromium (Cr), Cobalt (Co), Manganese (Mn), Zinc (Zn) does not attain the minimum statistical significant level when compared to controls. In addition, Aluminum was significantly increased in serum but decreased in CSF, may be due to the changes in organs other than CNS that are associated with PD. Overall, the analysis of data suggests significant overlap in CSF and serum for the elements Fe, Ca and Mg in PD, which demonstrates its potential application towards feasible diagnosis. In addition, the altered levels of these elements concentrations attribute changes in molecular mechanism of PD. For instance, brain requires a constant supply of Fe and diminished supply could result in neurological and cognitive dysfunction^10^. It has been observed that Fe influences oxidative stress by reducing hydrogen peroxide to release reactive oxygen species thereby causing severe oxidative damage^11^,^12^,^13^. Also, permeability of hydrogen peroxide across membranes that readily cause oxidative damage, which promotes mitochondrial dysfunction and neuronal damage^14^. In addition, significant association between decreased Fe and dopaminergic neurons were demonstrated in animal model^15^. Inconclusive results obtained from the previous studies of trace elements suggested decreased/no change in Fe levels among plasma/serum^5^,^16^,^17^,^18^. Alternatively, an increased Fe was noticed in *Lavanya et al.*^9^. However, the decreased concentration of serum Fe in the present study was in agreement with previous studies by Abbot *et al.*^19^, Ahmad *et al.*^20^. Also, data on CSF Fe corroborate with the earlier reports of Pall *et al.*^21^ and Jimenez-Jimenez *et al.*^22^, which was carried out on PD patients.

Our results also demonstrate the increased concentration of Ca and Mg in CSF and serum of PD patients. Calcium plays a vital role in neurotransmission, which triggers the release of neurotransmitter^23^. In addition, Ca regulates cytoplasm and nuclear calcium signals, which stimulate molecular pathways that promotes several transcription factors that are known to participate in synaptic plasticity. Few candidate genes of PD were identified to converge with the altered intracellular Ca. The DJ-1, PINK1, and LRRK2 genes have strongly implicated with Ca homeostasis^24^. In particular, DJ-1 protects against mitochondrial oxidant stress evoked by pacemaking in dopaminergic neurons, which interfere with mitochondrial uncoupling in response to calcium-induced stress^25^. Similarly, PINK1 contributes in maintaining of bioenergetic of mitochondria by regulating Ca efflux through Na+/Ca2+. Also, PINK1 was reported to cause mitochondrial Ca overload, resulting in mitochondrial oxidant stress^26^. Additionally, LRRK2 has been shown associated with Ca homeostasis, leading to mitochondrial depolarization and enhanced mitophagy, which can be prevented by L-type Ca^2+^ channel inhibitors^27^,^28^. Hence, calcium-channel blocker is suggested as the therapy for PD. Similarly, in our study Mg level was significantly increased in PD patients. Magnesium plays a vital role in cellular metabolism, signaling and synaptic neurotransmission. Mg has significant effects in neurotransmission of excitatory and inhibitory neurons^29^. In particular, Mg inhibits NMDA glutamate receptor that prevents the flow of ions at resting potentials^30^. High concentrations Mg block the calcium influx that restricts synaptic transmission, which decrease neuronal survival^31^. The increased Mg and Ca in PD serum were noticed in our study, which was in agreement with previous studies of Muralidhar *et al.*^7^ and Ahmed *et al.*^8^, that are associated with pathophysiology of PD.

Sub group analysis of PD treated and drug naive patients showed no significance difference in elemental concentration, suggesting the lack of influence of drugs in the elemental concentration in both CSF and Serum. Hence, the drug naive elemental data of serum and CSF were pooled with their treated groups for further analysis. In addition, the analysis based on clinical presentation, showed elements association of tremor-dominant and akinetic-rigidity of both CSF and serum. Further, the interdependency between the elements was demonstrated in CSF and serum using Pearson correlation. The results showed, the elements interactions patterns of normal and PD patients were similar in both the fluids although there were variations in certain interaction in PD patients. In CSF, Mn-Cr, Pb-Cr, Mg-Cr, Cu-Mn were altered, whereas Pb-Cu, Fe-Mg, Pb-Co, Zn-Al, Al-Co were altered in serum. This result showed the occurrence of altered interdependencies between the circulating elements of CSF and serum in PD, which reflects due to change in elemental homeostasis. Moreover, the multivariate analysis based on CSF and serum trace element data of normal and PD achieved a significant discrimination between groups. Surprisingly, significant discrimination in serum trace elements were noticed when compared to CSF.

To attain best and rapid method for PD diagnosis, machine learning algorithm was implemented. The success rate of classifiers using the trace element profile of CSF and serum data set was compared. According to multivariate analysis, serum showed best-performance in classifying the class and status when compared to algorithms trained with CSF data. Although serum is predicted to be a better medium in diagnosis of PD than CSF, which is much contrary to other studies, this might be due to incomparable sample sizes between the two biological media in the present study. Overall, it is suggested that trace element profile of serum could be potential in detecting and diagnosis of PD.

In conclusion, the present results of elements profiling, in PD compared to the control subjects confirmed the observations from previous study and identified common trace element signatures in CSF and serum of PD for feasible diagnosis. Interestingly, few elements demonstrated the fact of significant association with clinical presentation of PD, which helps in classifying patients based on clinical symptoms. Also, the patients on medications did not have an impact on element data with results of insignificant differences between treated and untreated groups. In addition to the elements analysis of serum and CSF, the implementation of neural network confirms the possibility of rapid detecting and diagnosis of PD. Surprisingly, serum showed relatively high accuracy compared to CSF in diagnosis. We believe the strength of the study is in utilizing neural network that apply elements changes in PD, which reduce complexity of diagnosis using MRI or CT. However, limitations in this study also warrant consideration. One of the major limitations of the study was CSF samples size, which is considerably small. The routine diagnosis of PD from CSF has several drawbacks: lumbar puncture and collection of CSF is an invasive treatment with potential side effects, and screening of patients is often difficult and follow-up analysis of the same patient over several years is problematic. Yet the role of trace elements in neurodegenerative diseases remains to clarify. Thus, prospective longitudinal studies concerning a regular follow-up procedure with larger number of subjects must be conducted to be able to establish definitive conclusions and throw of therapeutic behaviors to know a supplementation of some trace elements.

## Methods

### Clinical samples

The protocol of this study was approved by the Research Ethics Boards of the National Neuroscience Centre (NNC) and Nil Ratan Sircar Medical College and Hospital (NRS), Kolkata, India. This study was conducted in accordance with the approved protocols and the Declaration of Helsinki. Written informed consent was obtained from all participants. We enrolled 250 PD patients who were diagnosed by the neurologist (TKB and GG) of both the hospitals based on neurological examination and medical history. The patients were classified, based on their medical history and baseline evaluation, as having an akinetic-dominant and tremor-dominant. In order to have the number of patients with a true positive clinical diagnosis of PD at a maximum, UK Parkinson’s Disease Society Brain Bank Criteria, Unified Parkinson’s Disease Rating Scale (UPDRS)^32^ and Hoehn & Yahr scale^33^ were followed. All the cases have to meet the following symptoms of clinical classification of definite, probable, and possible PD at the time of diagnosis:

1. The presence of at least three of the following signs: resting tremor, cogwheel rigidity, bradykinesia and postural reflex impairment, at least one of which must be either rest tremor or bradykinesia. The disease has a unilateral onset and asymmetrical development, and the response to a dopaminergic agent is good to excellent.
2. No suggestion of secondary Parkinsonism due to drugs, trauma, brain tumor or treatment within the last 12 months with dopamine blocking or dopamine depleting agents and
3. No atypical features such as prominent occulomotor palsy, cerebellar signs, vocal cord paresis, severe orthostatic hypotension, pyramidal signs, amyotrophy or limb apraxia.

The following exclusion criteria were applied to both the patient and control groups:

- Ethanol intake higher than 80 g/day in the last 8 months.
- Previous history of chronic hepatopathy, chronic nephropathy or diseases causing malabsorption,
- Previous history of severe systemic disease
- Atypical dietary habits or under-nutrition
- Intake of copper, iron, aluminium, zinc, and chelating agents
- Therapy with cholorotiazides, ACTH, or steroids
- Acute infectious disorders, traumata, or surgery I the last 8 months,
- Haemolytic anemia

For comparative analysis, 280 age-gender matched healthy controls were recruited from the same hospitals. None of the controls had any neurological disorders, cognitive impairment or neuropsychiatric disability in their family history with similar educational levels. Every control individual underwent physical and laboratory examinations similar to the patients.

### Collection and storage of samples

CSF was collected from individuals in the acid-wash tube by lumbar puncture while lying on their side or in a seated position. Only 60 normal and 50 PD patients (subset of 250 PD patients contributed serum) consented to donate CSF. However, serum was collected from all the 530 individuals using sterile universal container without anticoagulant by standard venipuncture procedures. Both samples were immediately centrifuged, aliquoted and kept at −20C freezer for storage until the date of analysis. All the precautions were taken in accordance with NCCLS criteria (National committee for Clinical Laboratory Standards Approved Guidelines, 1997) to eliminate contamination while collecting and storing the samples. Further, samples were subjected to Atomic Absorption Spectrophotometry (SHIMADZU, AA-6200) and Flame Atomic Absorption Spectrophotometry (VARIAN AA-240, Varian Inc, USA) for element analysis. Atomic absorption spectrophotometry was executed to analyze Al, Co, Cr, Fe, Mn, Pb and Si in the samples, while Zn, Ca, Cu and Mg were determined using flame atomic absorption spectrophotometry. All calibration graphs were constructed using normal aqueous standards of NIST (National Institute for Standards and Technology, NIST, Gaithersburg, MD, USA).

### Statistical analysis

Statistical analysis was performed using SPSS version 16.0 software and R Version 3.3.0 (R Core Team, 2015)^34^ to determine the significant variations in elements concentration between PD and control subjects. For continuous variables (i.e., age and elements), testing the location difference between the PD and control groups was determined by using the Wilcoxon rank-sum test. The null hypothesis is that the location parameters of the PD and control groups are the same while the alternative hypothesis is that the location parameters of the two groups are different (i.e., two-sided hypothesis). For binary variable (i.e., gender), testing the difference between the gender proportions of the PD and control groups was done by using the two-sample test for population proportions. Additionally, the Pearson correlation and element ratio analysis were implemented to establish interdependency between elements among normal and patient samples. Further, multivariate analysis was carried out to reduce the multi-dimensionality of the element data and to reveal grouping ability of CSF and serum data as normal and PD^35^. Also, the analysis was extended, to assess cross sectional variations in both CSF and serum samples with different clinical factors of PD, which describes the association between elements concentrations and clinical presentations of Parkinson’s disease. The orthogonal partial least square discriminant analysis (OPLS-DA) is performed by using the “devium” package in R. For the 530 subjects who contributed serum for the study, first it was calculated a large (10 latent variables (LV)) exploratory model for 0 and 1 orthogonal latent variables (O-LVs).

### Neural network Algorithm

The machine learning algorithms were used to determine the efficiency in classification of disease from normal using trace element data of CSF and serum. The Weka software (Waikato Environment for Knowledge Analysis) was implemented for neural network^35^, and evaluation of algorithms such as Bayes Net, JRip, Multilayer Perceptron, Naive Bayes, RBF Network, Random Forest and Simple Logistic were done with ten times, ten-fold cross-evaluation. This method uses four-fifths of data for training the model precision, recall, accuracy F-measure and Area under ROC.


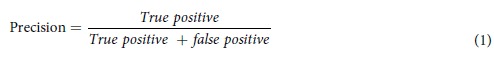



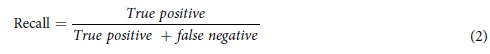



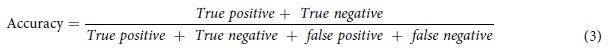



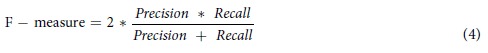


## Additional Information

**How to cite this article**: Sanyal, J. *et al.* Metallomic Biomarkers in Cerebrospinal fluid and Serum in patients with Parkinson’s disease in Indian population. *Sci. Rep.* **6**, 35097; doi: 10.1038/srep35097 (2016).

## Supplementary Material

Supplementary Information

## Figures and Tables

**Table 1:** Demographic and clinical characteristics of PD and control subjects (serum and CSF).

Clinical parameters	Serum			CSF		
	Control Average ± SD	PD Average ± SD	*p*-value	Control Average ± SD	PD Average ± SD	*p*-value
Age (years)	56.42 ± 9.68	57.88 ± 12.059	0.323	60.11 ± 10.44	58.72 ± 12.38	0.534
Gender ratio (Male:Female)	183:97	162:88	0.965	42:18	34:16	0.985
Age at Onset (years)	—	53.1 ± 11.2yrs		—	54.91 ± 13.2yrs	
Disease duration (years)	—	4.92 ± 3.04		—	3.77 ± 2.3	
Total for parts I-III (items 1–31)	—	31.2 ± 5.2		—	26.82 ± 7.94	
ADLs scale (items 5–17)	—	13.8 ± 1.8		—	12.5 ± 1.3	
Motor scale (items 18–31)	—	14.9 ± 2.1		—	11.7 ± 1.6	
Hoehn and Yahr stage	—	2.4 ± 1.1		—	2.16 ± 1.09	

**Table 2:** Elements concentration in CSF and serum of PD versus control.

Elements	CSF Concentration Average (±SD)			Serum Concentration Average (±SD)		
	Normal	PD	*p*-value	Normal	PD	*p*-value
Al	2.9 (±0.84)	2.45 (±1.47)	0.1701*	3.14 (±1.94)	4.29 (±2.06)	<0.00001*
Ca	25,921 (±5533)	28,130 (±3409)	0.03697*	65392.97 (±1755)	710950 (±2177)	<0.00001*
Co	0.12 (±0.093)	0.07 (±0.13)	<0.0001*	0.15 (±0.27)	0.14 (±0.29)	<0.00001
Cu	27.36 (±5.1	24.89 (±7.92)	0.03862*	1099 (±114.85)	891.82 (±197.93)	<0.00001*
Cr	0.98 (±0.63)	1.22 (±0.41)	0.04784*	0.71 (±0.72)	0.83 (±0.83)	0.07121
Fe	212.46 (±33.58)	182.88 (±89.28)	0.000323*	1205.49 (±316.09)	1155.55 (±264.94)	0.01231*
Pb	0.53 (±0.82)	0.97 (±0.98)	0.000801*	0.41 (±0.21)	0.49 (±0.37)	0.1161*
Mg	22,739.05 (±2386)	25,263 (±3433)	0.000114*	19883.8 (±1480.82)	20403.71 (±1244.19)	0.0002269*
Mn	1.07 (±0.81)	0.78 (±0.65)	0.08653*	1.35 (±1.17)	1.28 (±1.02)	0.3643
Si	83.69 (±35.64)	69.59 (±29.35)	0.08221*	402.6 (±105.73)	386.38 (±99.63)	0.02221
Zn	30.73 (±7.91	27.17 (±8.07	0.03259*	700.15 (±86.73)	687.39 (±81.14)	0.01592

**Table 3:** Elements concentration in CSF and serum of un-medicated PD versus medicated PD.

Elements	CSF Concentration Average (±SD)			Serum Concentration Average (±SD)		
	umPD	mPD	*p*-value	umPD	mPD	*p*-value
Al	1.68 (±1.21)	1.66 (±1.08)	0.8857	4.26 (±1.66)	3.78 (±1.68)	0.1419
Ca	28904.55 (±2918.05)	26199.17 (±6912.84)	0.3429	71395.96 (±2041.19)	71412.9 (±1958.48)	0.7798
Co	0.02 (±0.02)	0.25 (±0.28)	0.4857	0.09 (±0.16)	0.11 (±0.29)	0.8082
Cu	20.15 (±7.02)	28.02 (±3.39)	0.2000	898.53 (±264.30)	886.54 (±223.01)	0.4016
Cr	1.49 (±0.29)	1.24 (±0.49)	0.4857	0.85 (±0.89)	0.62 (±0.63)	0.2258
Fe	195.5 (±115.27)	156.75 (±45.53)	1.0000	1125.67 (±371.94)	1128.58 (±291.87)	0.4085
Pb	0.61 (±0.70)	1.04 (±1.23)	0.6857	0.51 (±0.35)	0.46 (±0.32)	0.4911
Mg	23730.17 (±3543.33)	24395.28 (±3003.13)	0.6857	20593.87 (±1514.19)	20247.17 (±1043.04)	0.2320
Mn	0.99 ((±0.81)	0.92 (±0.70)	0.8845	1.38 (±1.16)	1.25 (±1.11)	0.6986
Si	75.51 ((±33.04)	71.78(±39.88)	1.0000	384.81 (±84.22)	392.94 ((±89.73)	0.7054
Zn	24.57 (±6.42)	22.60 (±6.89)	0.8857	696.35 (±65.49)	688.18 (±110.94)	0.7032

**Table 4:** Elements concentration in CSF and serum of akinetic-dominant PD versus control.

Elements	CSF Concentration Average (±SD)			Serum Concentration Average (±SD)		
	Normal	PD	*p*-value	Normal	PD	*p*-value
Al	2.97 (±0.92)	2.56 (±1.58)	0.4889	2.97 (±2.27)	4.19 (±1.99)	<0.00001*
Ca	27372.0 (±3295.0)	28170.8 (±2633.7)	0.5946	65314.1 (±1899.8)	71063.7 (±2327.0)	<0.00001*
Co	0.085 ± 0.11	0.047 (±0.092)	<0.00001*	0.17 (±0.28)	0.13 (±0.22)	0.0281*
Cu	30.33 (±3.46)	24.59 (±7.039)	0.0026*	1066.54 (±130.65)	882.63 (±189.10)	<0.00001*
Cr	1.23 (±0.28)	1.20 (±0.36)	0.9309	0.60 (±0.54)	0.73 (±0.74)	0.17
Fe	214.54 (±43.81)	206.37 (±101.13)	0.2394	1260.82 (±300.91)	1169.84 (±267.19)	<0.00001*
Pb	0.87 (±0.72)	0.99 (±0.96)	0.7548	0.35 (±0.209)	0.53 (±0.38)	0.0002*
Mg	21899.3 (±1950.8)	25174.8 (±3651.9)	0.00095*	19715.0 (±1397)	20489.7 (±1401.1)	0.0004*
Mn	0.74 (±0.64)	0.73 (±0.63)	0.9931	1.33 (±0.94)	1.30 (±1.10)	0.4089
Si	70.01 (±24.38)	78.37 (±27.75)	0.06046	416.62 (±120.50)	389.41 (±104.18)	0.0341*
Zn	27.00 (±8.42)	26.43 (±8.56)	0.7228	685.50 (±96.17)	685.09 (±90.56)	0.9010

**Table 5:** Elements concentration in CSF and serum of tremor-dominant PD versus control.

Elements	CSF Concentration Average (±SD)			Serum Concentration Average (±SD)		
	Normal	PD	*p*-value	Normal	PD	*p*-value
Al	2.90 (±0.90)	2.46 (±1.53)	0.2403	3.22 (±1.57)	4.21 (±2.03)	<0.00001*
Ca	25212.3 (±6545.3)	27847.2 (±3424.4)	0.03579*	65486.6 (±1774.7)	70897.4 (±2207. 8)	<0.00001*
Co	0.15 (±0.07)	0.07 (±0.12)	<0.00001*	0.14 (±0.25)	0.13 (±0.30)	0.0748
Cu	25.56 (±5.06)	25.96 (±8.33)	0.9834	1109.33 (±97.50)	894.30 (±196.08)	<0.00001*
Cr	0.82 (±0.73)	1.20 ((±0.43)	0.0191*	0.77 (±0.77)	0.88 (±0.87)	0.1911
Fe	211.32 (±23.42)	187.29 (±89.49)	0.0164*	1146.58 (±299.57)	1137.33 (±260.59)	0.05455
Pb	0.21 (±0.74)	1.01 (±0.95)	<0.00001*	0.44 (±0.24)	0.50 (±0.39)	0.6181
Mg	23092.8 (±2524.9)	25302.9 (±3375.4)	0.00288*	19977.2 (±1375.4)	20510.1 (±1227.6)	0.00026*
Mn	1.27 (±0.90)	0.75 (±0.67)	0.00232*	1.41 (±1.10)	1.31 (±1.12)	0.1934
Si	95.41 (±39.66)	72.63 (±29.26)	0.01711*	386.99 (±96.01)	389.42 (±101.50)	0.6105
Zn	32.69 (±6.75)	26.27 (±8.25)	0.00078*	693.58 (±89.23)	689.43 (±85.31)	0.2312

**Table 6:** Neural network prediction using A) CSF data and B) serum data.

A)	BayesNet	NaiveBayes	SimpleLogistic	RBFNetwork	JRip	Random Forest	Multilayer Perceptron
**CSF Class Prediction** (Normal and Parkinson’s disease)							
**Accuracy**	99.78	98.18	96.72	98.63	94.54	98.63	97.45
**Precision**	1	0.9	1	0.9	0.9	0.9	1
**Recall**	0.9	0.9	0.9	0.9	0.9	0.9	0.9
**F-measure**	0.9	0.9	0.9	0.9	0.9	0.9	0.9
**Area under ROC**	1	0.9	0.9	0.9	0.9	0.9	0.9
**CSF Stages Prediction** (normal, stage1, stage2 and stage3 PD)							
**Accuracy**	71.73	68.64	70.73	69.36	67.18	70.36	68.91
**Precision**	0.6	0.4	0.5	0.3	0.4	0.5	0.4
**Recall**	0.7	0.5	0.5	0.6	0.4	0.6	0.5
**F-measure**	0.7	0.4	0.5	0.4	0.4	0.5	0.4
**Area under ROC**	0.8	0.8	0.8	0.7	0.6	0.8	0.8
**CSF Status Prediction **(Normal, Progressive and Static)							
**Accuracy**	79.72	82.45	83.72	83.27	80.90	84.09	82.27
**Precision**	0.7	0.7	0.7	0.7	0.7	0.7	0.7
**Recall**	0.7	0.6	0.8	0.8	0.7	0.8	0.7
**F-measure**	0.7	0.7	0.7	0.7	0.74	0.7	0.7
**Area under ROC**	0.9	0.9	0.9	0.8	0.8	0.9	0.9
B) **Serum Class Prediction **(Normal and Parkinson’s disease)							
**Accuracy**	99.87	98.63	99.67	98.80	94.54	96.72	99.78
**Precision**	0.9	0.9	0.9	0.9	0.9	1	0.9
**Recall**	1	0.9	1	1	0.9	0.9	1
**F-measure**	0.9	0.9	0.9	0.9	0.9	0.9	0.9
**Area under ROC**	1	0.9	1	0.9	0.9	0.9	1
**Serum Stages Prediction** (normal, stage1, stage2 and stage3 PD)							
**Accuracy**	65.06	69.28	68.46	69.16	72.95	67.33	68.29
**Precision**	0.7	1	0.9	0.9	1	0.9	0.9
**Recall**	0.9	1	0.9	1	1	0.9	1
**F-measure**	0.8	1	0.9	0.9	1	0.9	0.9
**Area under ROC**	0.8	1	1	0.9	1	0.9	0.9
**Serum Status Prediction **(Normal, Progressive and Static)							
**Accuracy**	88.64	87.91	90.57	88.89	87.95	88.56	91.07
**Precision**	0.8	1	0.9	0.9	0.9	0.9	0.9
**Recall**	0.8	0.9	1	1	0.9	0.9	1
**F-measure**	0.7	0.9	0.9	0.9	0.9	0.9	0.9
**Area under ROC**	0.8	0.8	0.9	0.9	0.9	0.9	0.9
