# Supplementary material for: Metallomic Biomarkers in Cerebrospinal fluid and Serum in patients with Parkinson’s disease in Indian population
Source: Sci Rep. 2016 Oct 18;6:35097. doi: 10.1038/srep35097 (PMC5067653; doi:10.1038/srep35097)
Supplement: Supplementary Information [file srep35097-s1.doc]

**Metallomic Biomarkers in Cerebrospinal fluid and Serum in patients with Parkinson’s disease in Indian population**

Jaya Sanyal^a#^, Shiek SSJ Ahmed^b#^, Hon Keung Tony Ng^c^, Tufan Naiya^d^, Epsita Ghosh^e^, Tapas Kumar Banerjee^e^, Jaya Lakshmi^f^, Gautam Guha^g^, Vadlamudi Raghavendra Rao^a^*

**^a^** Department of Anthropology, University of Delhi, Delhi-110007, INDIA.

**^b^** Drug Discovery Lab, Faculty of Allied Health Sciences, Chettinad Academy of Research and Education, Tamil Nadu, INDIA.

**^c^** Department of Statistical Science, Southern Methodist University, Dallas, TX 75275, USA

^d^ Molecular Systematics Division, Zoological Survey of India, DNA Taxonomy Laboratory, Kolkata, INDIA.

^e^ Department of Neurology, National Neurosciences Centre, Kolkata, INDIA.

^f^Dept.of Life Sciences, Central University Of Tamil Nadu, Thiruvarur,Tamil Nadu, INDIA.

^g^Department of Neurology, Nil Ratan Sircar Medical College and Hospital, Kolkata, INDIA.

^#^Jaya Sanyal and ^#^Shiek SSJ Ahmed are joint first authors as they equally contributed towards the manuscript

**Corresponding author:**

Prof. V. R. Rao

Department of Anthropology

University of Delhi, North Campus,

New Delhi-110007, INDIA

E-mail Id: parkinsons_research@rediffmail.com

**Supplementary Table 1. a) Serum Element inter-relationship in PD for element linkage analysis**

| **Correlation between elements (r)** | | | |
| --- | --- | --- | --- |
| **Element X** | **Element Y** | **Degree of correlation in PD** | **Degree of correlation in controls** |
| Ca | Mg | 0.042 | 0.015 |
| Cu | Zn | 0.201* | 0.056 |
| Cu | Ca | -0.034 | -0.083 |
| Cu | Pb | -0.158* | 0.135* |
| Cu | Fe | 0.056 | -0.100 |
| Cu | Cr | -0.019 | -0.045 |
| Fe | Mn | 0.215* | 0.002 |
| Fe | Pb | -0.243* | -0.092 |
| Fe | Zn | 0.147* | 0.063 |
| Fe | Co | 0.196* | 0.111 |
| Fe | Ca | -0.035 | 0.049 |
| Fe | Mg | -0.016 | -0.234* |
| Fe | Cr | 0.055 | -0.051 |
| Mn | Zn | -0.107 | -0.011 |
| Mn | Ca | 0.043 | 0.101 |
| Mn | Pb | -0.025 | -0.033 |
| Mn | Cr | 0.101 | -0.106 |
| Mn | Co | -0.008 | -0.092 |
| Pb | Ca | 0.186* | 0.0145 |
| Pb | Cr | 0.184* | 0.046 |
| Pb | Co | 0.036 | 0.152* |
| Zn | Pb | 0.035 | -0.003 |
| Zn | Ca | -0.006 | 0.0786 |
| Zn | Mg | 0.171* | -0.0005 |
| Mg | Cu | 0.035 | 0.052 |
| Zn | Al | -0.0003 | -0.178* |
| Zn | Co | -0.079 | -0.028 |
| Zn | Cr | -0.036 | 0.050 |
| Mg | Cr | 0.063 | 0.024 |
| Mg | Al | 0.151* | 0.138 |
| Mg | Pb | -0.109 | -0.080 |
| Mg | Mn | 0.087 | 0.087 |
| Mg | Co | -0.068 | -0.057 |
| Ca | Cr | 0.122 | -0.011 |
| Ca | Al | 0.071 | 0.0111 |
| Ca | Co | -0.012 | -0.022 |
| Al | Mn | 0.162* | 0.08 |
| Al | Pb | -0.134 | 0.019 |
| Al | Fe | 0.077 | -0.102 |
| Al | Cu | 0.159* | 0.112 |
| Al | Co | 0.026 | -0.169* |
| Al | Cr | 0.035 | 0.045 |
| Cu | Co | 0.0003 | 0.006 |
| Cu | Mn | -0.059 | -0.047 |
| Cr | Co | 0.165* | 0.001 |
| Si | Al | 0.725 | 0.105 |
| Si | Ca | -0.149* | 0.002 |
| Si | Co | -0.002 | 0.038 |
| Si | Cr | 0.007 | 0.014 |
| Si | Cu | 0.101 | -0.050 |
| Si | Fe | -0.029 | 0.080 |
| Si | Mg | -0.038 | -0.152* |
| Si | Mn | -0.104 | -0.047 |
| Si | Pb | -0.104 | 0.014 |
| Si | Zn | -0.004 | -0.076 |
| **1b) CSF Element inter-relationship in PD for element linkage analysis** | | | |
| Ca | Mg | -0.107 | 0.071 |
| Cu | Zn | -0.155 | -0.129 |
| Cu | Ca | -0.186 | 0.243 |
| Cu | Pb | 0.356* | 0.025 |
| Cu | Fe | 0.145 | -0.073 |
| Cu | Cr | -0.138 | 0.055 |
| Fe | Mn | -0.033 | 0.068 |
| Fe | Pb | 0.2 | -0.225 |
| Fe | Zn | -0.422* | -0.148 |
| Fe | Co | -0.245 | -0.122 |
| Fe | Ca | 0.077 | 0.136 |
| Fe | Mg | 0.173 | -0.151 |
| Fe | Cr | -0.030 | -0.071 |
| Mn | Zn | 0.074 | 0.015 |
| Mn | Ca | 0.111 | -0.150 |
| Mn | Pb | 0.109 | -0.002 |
| Mn | Cr | 0.240 | 0.320* |
| Mn | Co | 0.286 | 0.188 |
| Pb | Ca | 0.139 | -0.131 |
| Pb | Cr | -0.059 | 0.450* |
| Pb | Co | 0.151 | -0.151 |
| Zn | Pb | -0.139 | -0.179 |
| Zn | Ca | 0.293* | -0.115 |
| Zn | Mg | 0.034 | 0.113 |
| Mg | Cu | 0.219 | -0.042 |
| Zn | Al | 0.371 | 0.106 |
| Zn | Co | 0.075 | 0.036 |
| Zn | Cr | -0.113 | -0.162 |
| Mg | Cr | -0.392* | -0.306* |
| Mg | Al | -0.137 | 0.121 |
| Mg | Pb | 0.266 | -0.132 |
| Mg | Mn | -0.233 | -0.033 |
| Mg | Co | -0.049 | 0.095 |
| Ca | Cr | 0.069 | -0.152 |
| Ca | Al | 0.159 | 0.027 |
| Ca | Co | 0.131 | -0.079 |
| Al | Mn | 0.080 | 0.003 |
| Al | Pb | -0.198 | -0.064 |
| Al | Fe | -0.211 | 0.094 |
| Al | Cu | -0.234 | -0.052 |
| Al | Co | 0.0012 | 0.046 |
| Al | Cr | 0.085 | 0.114 |
| Cu | Co | 0.193 | -0.084 |
| Cu | Mn | -0.249 | -0.418* |
| Cr | Co | 0.008 | -0.124 |
| Si | Al | -0.235 | 0.122 |
| Si | Ca | -0.236 | -0.493* |
| Si | Co | -0.372* | 0.189 |
| Si | Cr | 0.020 | 0.261* |
| Si | Cu | 0.110 | -0.251 |
| Si | Fe | 0.333* | -0.062 |
| Si | Mg | -0.192 | -0.086 |
| Si | Mn | -0.116 | 0.494* |
| Si | Pb | -0.012 | -0.071 |
| Si | Zn | -0.528* | -0.056 |

- *p<0.05, (significance)*

**Supplementary Table 2. C/B ratios of trace elements among patients and controls**

|  | **Patient** | **Control** |
| --- | --- | --- |
| **Iron** | 0.162 | 0.176 |
| **Copper** | 0.027 | 0.024 |
| **Calcium** | 0.395 | 0.396 |
| **Aluminium** | 0.78 | 0.461 |
| **Magnesium** | 1.238 | 1.143 |
| **Manganese** | 0.609 | 0.792 |
| **Silicon** | 0.179 | 0.207 |
| **Zinc** | 0.039 | 0.043 |
| **Lead** | 1.979 | 1.292 |
| **Cromium** | 1.457 | 1.38 |
| **Cobalt** | 0.5 | 0.8 |

**Supplementary Figure Legends:**

Supplementary Figure S1. Elements concentrations in CSF of 60 normal and 50 PD patients, represented as heat map, green represent down regulation and red color as up regulation.

Supplementary Figure S2. Elements concentration in serum among 280 normal and 250 PD patients, represented as heat map.

Supplementary Fig. S3. Number of latent variables in the OPLS-DA model verse root mean squared error of prediction based on the serum data.

Supplementary Fig. S4. Number of latent variables in the OPLS-DA model verse root mean squared error of prediction based on the CSF data.


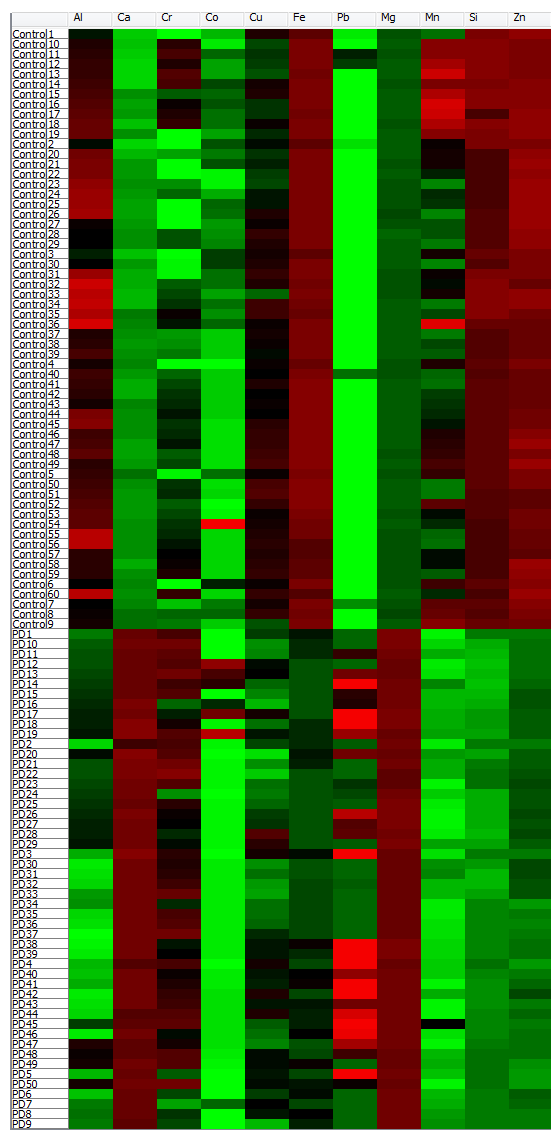

Supplementary Figure S1. Elements concentrations in CSF of 60 normal and 50 PD patients, represented as heat map, green represent down regulation and red color as up regulation.


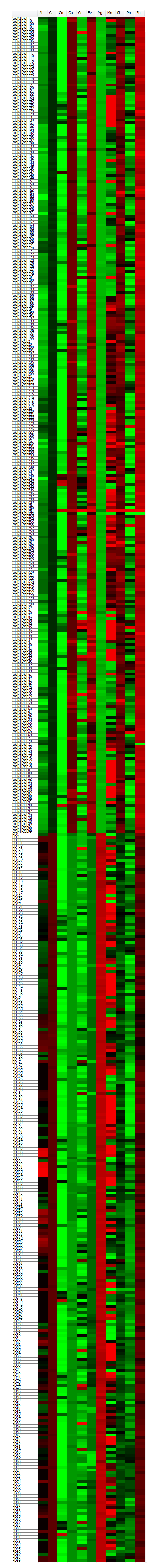

Supplementary Figure S2. Elements concentration in serum among 280 normal and 250 PD patients, represented as heat map.


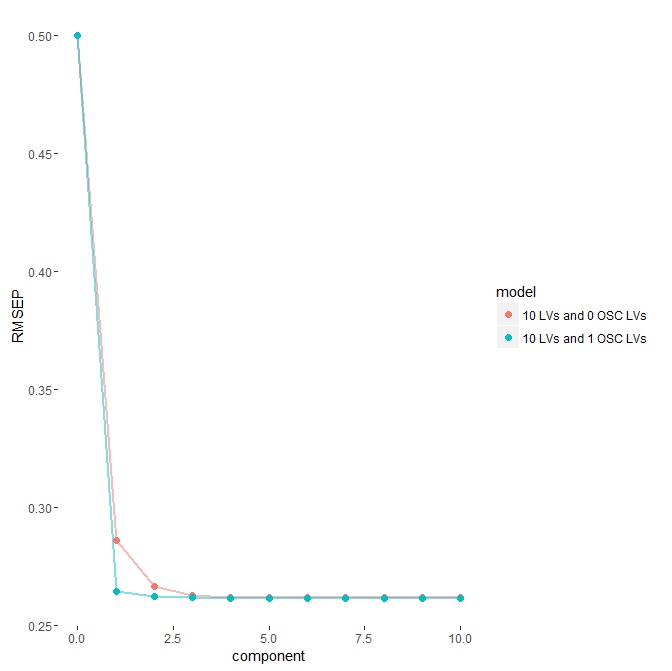


Supplementary Fig. S3. Number of latent variables in the OPLS-DA model verse root mean squared error of prediction based on the serum data.


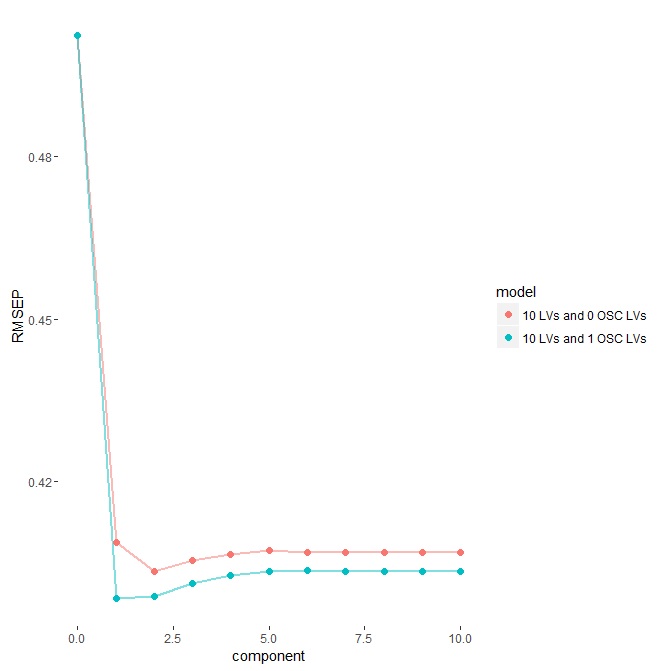


Supplementary Fig. S4. Number of latent variables in the OPLS-DA model verse root mean squared error of prediction based on the CSF data.
